# Comparison of cold snare polypectomy for sessile serrated lesions ≥10 mm between experienced and trainee endoscopists: A propensity score matching cohort study

**DOI:** 10.1002/deo2.328

**Published:** 2024-01-06

**Authors:** Yoshiaki Kimoto, Rikimaru Sawada, Susumu Banjoya, Toshihumi Iida, Tomoya Kimura, Koichi Furuta, Shinya Nagae, Yohei Ito, Hiroshi Yamazaki, Nao Takeuchi, Syunya Takayanagi, Yuki Kano, Takashi Sakuno, Kohei Ono, Ryoju Negishi, Eiji Sakai, Yohei Minato, Hideyuki Chiba, Ken Ohata

**Affiliations:** ^1^ Department of Gastrointestinal Endoscopy NTT Medical Center Tokyo Tokyo Japan; ^2^ Division of Gastroenterology Itabashi Chuo Medical Center Tokyo Japan; ^3^ Division of Gastroenterology Yokohama Sakae Kyosai Hospital Kanagawa Japan; ^4^ Department of Gastroenterology Omori Red Cross Hospital Tokyo Japan

**Keywords:** cold snare polypectomy, sessile serrated lesion, endoscopic submucosal dissection, piecemeal cold snare polypectomy, endoscopic mucosal resection

## Abstract

**Objectives:**

Previous studies of cold snare polypectomy (CSP) for sessile serrated lesions (SSLs) ≥10 mm were performed by experienced endoscopists, and therefore their skills might have significantly influenced results. In this study, we compared the efficacy and safety of CSP for SSLs ≥10 mm between experienced and trainee endoscopists.

**Methods:**

In a 1:1 propensity score matched retrospective cohort study, we compared the complete resection rate, en‐bloc resection rate, adverse event rate, and procedure time between experienced and trainee groups. Thirteen endoscopists performed CSP, and we defined the experienced group as endoscopists with board certification from the Japan Gastroenterological Endoscopy Society.

**Results:**

We examined 616 lesions with SSLs ≥10 mm resected by CSP between February 2018 and May 2022. We excluded 61 lesions from the analysis because they had simultaneously undergone hot snare polypectomy (*n* = 57) or had been taken over by experienced endoscopists from trainees in the CSP procedure (*n* = 4). Finally, we identified 217 propensity score‐matched pairs (*n* = 434). Between experienced and trainee groups, the results were complete resection rate (100 vs. 100%; *p =* 1.00), en‐bloc resection rate (73.2 vs. 75.6%; *p =* 0.24), adverse event rate (3.2 vs. 2.8%; *p =* 0.77), or procedure time (6.2 vs. 5.9 min; *p =* 0.64).

**Conclusions:**

We have demonstrated the safety and efficacy of CSP for SSLs ≥10 mm performed by experienced and trainee endoscopists.

## INTRODUCTION

The risk of sessile serrated lesions (SSLs) progressing to cancer is low, regardless of tumor size, when compared with that of conventional adenomas.^1^, ^2^, ^3^ Various endoscopic treatments have been applied to SSLs as secondary prevention to reduce the mortality rate of colorectal cancer.^4^, ^5^ Endoscopic mucosal resection (EMR) and endoscopic submucosal dissection (ESD) are associated with a risk of serious adverse events, such as clinically significant delayed post‐polypectomy bleeding (DPPB) and perforation, even when the procedures are performed by experienced endoscopists.^6^, ^7^ Adverse events may require unscheduled hospitalization. In addition, multiple SSLs may require consecutive colonoscopies and multiple hospitalizations to remove all the lesions, increasing patient burden. On the other hand, although cold snare polypectomy (CSP) has been reported to be a safe and effective treatment for non‐pedunculated colonic polyps less than 10 mm in size,^8^, ^9^ relatively few reports have discussed CSP for SSLs ≥10 mm.

Recently, studies of CSP or piecemeal CSP (pCSP) for SSLs ≥10 mm have been reported,^10^, ^11^, ^12^, ^13^, ^14^, ^15^, ^16^ but no studies have examined the impact of endoscopists’ experience on the outcomes of CSP or pCSP for SSLs ≥10 mm. Most of these reports were performed by experienced endoscopists. Therefore, the technical skills of the endoscopists might have had a significant impact on our previous results; CSP for SSLs ≥10 mm might require a level of endoscopy experience similar to that required for EMR and ESD.^7^ Thus, whether CSP for SSLs should be performed only by an experienced endoscopist, as with colorectal ESD, or whether this procedure should only be performed at high volume centers remains uncertain.

In the present study, we assessed the efficacy and safety of CSP for SSLs ≥10 mm when performed by both experienced and trainee endoscopists.

## METHODS

### Study design

This study was designed as a single‐center, retrospective, cohort study to investigate the efficacy and safety of CSP for SSLs ≥10 mm when performed by experienced and trainee endoscopists. This study was approved by the ethics committee of the NTT Medical Center Tokyo (No. 18–81).

### Patients and lesions

We examined the maintained database of NTT Medical Center Tokyo and identified 616 consecutive lesions (393 patients) with SSLs ≥10 mm resected by CSP between February 2018 and May 2022. Lesions recruited from the previous study were also included in this report to assess the influence of endoscopists.^10^ Lesions with the following endoscopic findings associated with cytological dysplasia or cancer were excluded^3^, ^4^: (i) double elevation, (ii) central depression, (iii) reddishness, and (iv) a Japan narrow‐band imaging Expert Team type 2/3 surface or vessel pattern. We also excluded lesions that had simultaneously undergone a hot snare polypectomy, such as EMR or polypectomy. Furthermore, we excluded lesions that had been taken over by experienced endoscopists from trainees in the CSP procedure.

### CSP procedure and perioperative management

All patients underwent standard bowel cleansing with 2 L of polyethylene glycol. The bowel preparation scale in the polyp‐bearing segment was documented using the Aronchick scale.^17^ Before CSP, we performed endoscopic diagnoses using not only standard white light, but also chromoendoscopy with 0.2% indigo carmine or image enhancement endoscopy performed using narrow‐band imaging (Olympus) or blue light imaging (Fujifilm Corporation) or optical enhancement (Pentax Medical) to exclude SSLs with cytological dysplasia or cancer. We performed the CSP procedures using lower gastrointestinal endoscopes (e.g., models PCF‐Q260AZI and PCF‐H290ZI, Olympus; models EC‐L600ZP7 and EC‐L600MP7, Fujifilm Corporation; model EC‐3890MZi, Pentax Medical) equipped with a black attachment cap (MB‐46; Olympus) and using carbon dioxide insufflation. The morphology was classified according to the Paris classification.^18^

**FIGURE 1:**
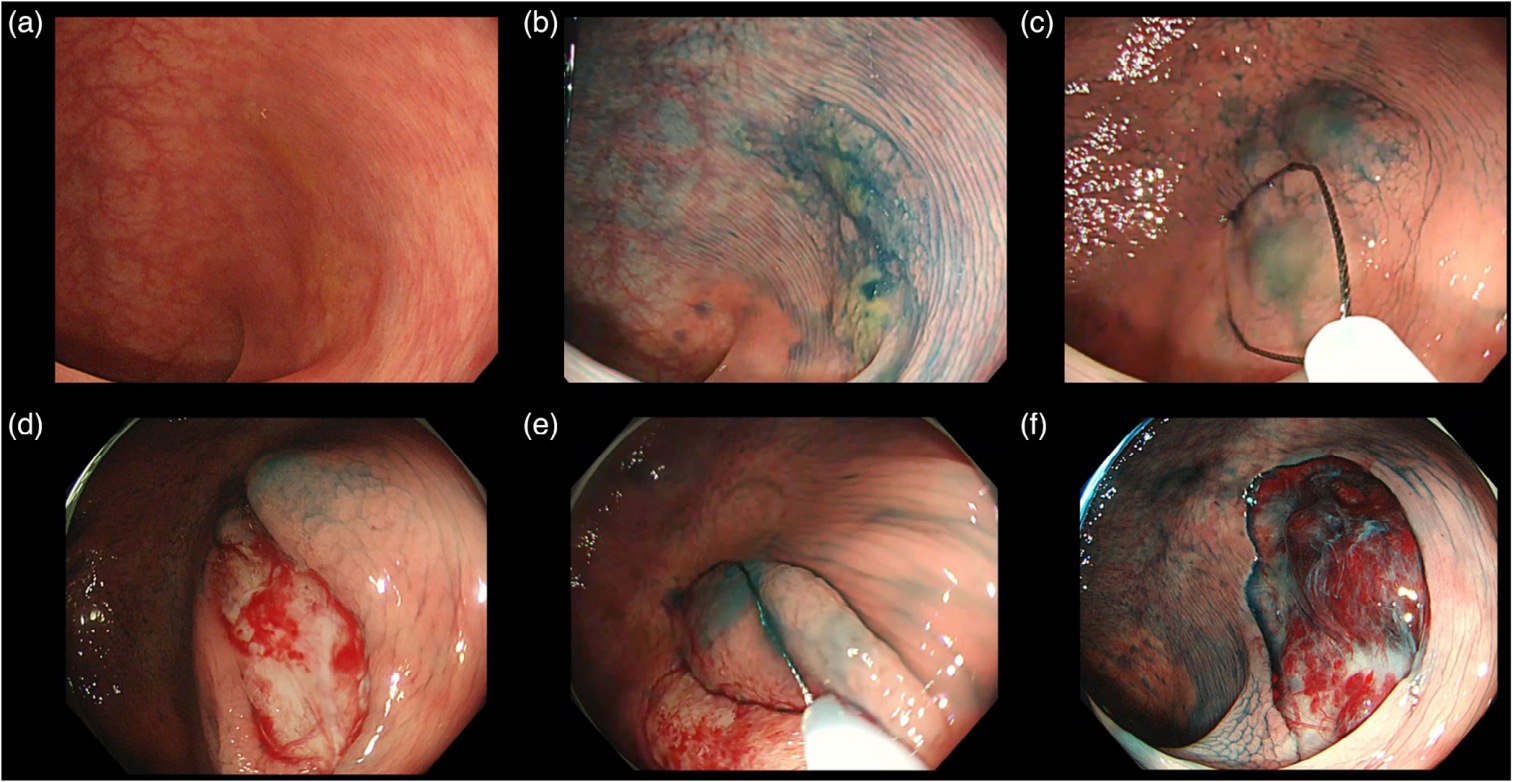
Piecemeal cold snare polypectomy (pCSP). (a) A flat elevated 25‐mm lesion was located in the transverse colon. (b) Before pCSP, the endoscopic diagnosis was made by spraying the lesion with 0.2% diluted indigo carmine, in addition to white light observation. (c) The 1st time snaring without injection using a 10‐mm oval snare. (d) After the 1st time snaring. (e) The 2nd time snaring without injection using a 10‐mm oval snare. (f) Complete resection was archived with 3‐time piecemeal resection. The lesion together with a 2–3 mm margin of normal tissue was removed with a snare designed for pCSP procedures.

All CSP procedures were performed without submucosal injection. Using a snare designed for CSP (Dualoop, Medico's Hirata Inc.; width, 12 mm; length, 25 mm; or SnareMaster Plus, Olympus; width, 10 mm; length, 20 mm), each lesion was resected together with a 2–3 mm margin of normal tissue. We performed pCSP in certain larger SSLs when an en‐bloc resection was difficult. The pCSP procedure has been previously described in detail^10^, ^19^ and is shown in Figure 1. pCSP was used to resect large lesions together with a normal tissue margin of 2–3 mm. We carefully evaluated the margins by staining with indigo carmine and performing image enhancement endoscopy after resection, then sampled the margins of the defect using biopsy forceps (EndoJaw; Olympus) according to the following protocol. In an en‐bloc resection, two‐point biopsies were performed from two marginal sites located symmetrically in the mucosal defects, while in pCSP, circular four‐point biopsies were performed. We did not perform tattooing because the presence of multiple SSLs is relatively common and the difficulty of future treatment would likely be increased as a result of fibrosis if an SSL were to appear on the tattooed area. Therefore, we thoroughly described the distance from the anal verge and the location of the lesion in each report. We determined the location of the lesion based on the anatomic features of each colon segment. No prophylactic clipping without immediate postpolypectomy bleeding (IPB) was performed. All lesions were classified according to the WHO classification system.^20^

### Endoscopists

Thirteen endoscopists performed the CSP procedures. In this study, we defined an experienced endoscopist as an endoscopist with board certification from the Japan Gastroenterological Endoscopy Society. This certification requires 5 years of training in board‐certified institutions of the Japan Gastroenterological Endoscopy Society and experience in therapeutic colonoscopies, furthermore, it is mandatory to pass the written test. In this study, eight of the 13 endoscopists fell into an experienced group, and they all had performed more than 2000 colonoscopies and more than 50 colorectal ESD. On the other hand, five of the 13 endoscopists were not yet certified by the Japan Gastroenterological Endoscopy Society, all of them had been endoscopists for less than 3 years, had performed less than 2000 colonoscopies, and had never performed colorectal ESD. When an experienced endoscopist took over from a trainee in the CSP procedure, that lesion was excluded from the analysis. Based on this definition, we divided the subjects into experienced and trainee groups and examined the efficacy and safety of CSP for SSL of ≥10 mm for each of these groups.

### Outcome measurements

The outcomes were the complete resection rate, en‐bloc resection rate, adverse event rate, and procedure time. We defined a complete resection as a negative biopsy of the post‐CSP mucosal defect. We defined IPB as spurting or oozing lasting more than 30 seconds, while DPPB was defined as a hemorrhage requiring an endoscopic hemostatic procedure and occurring within 28 days after CSP. All patients underwent follow‐up outpatient visits within 28 days after CSP and were checked for the presence of adverse events. The procedure time for CSP was measured from the insertion of the snare into the working channel until the completion of the treatment. Surveillance colonoscopy (SC) was recommended for all patients enrolled in this study.

### Statistical analysis

To reduce the effects of selection bias and potential confounders, we performed propensity score matching with a 1:1 ratio using nearest‐neighbor matching without replacement within a caliper width at a 0.2 standard deviation of the propensity score. We compared the following variables between experienced and trainee groups: sex (male or female), age, colon preparation (good‐excellent or fair), previous endoscopic attempt including a biopsy of the target lesion (yes or no), size, location (proximal or distal colon), morphology (type 0‐I or 0‐II) and antithrombotic therapy (continuation or none). The proximal colon was defined as colon segments proximal to the splenic flexure, and the distal colon was defined as colon segments distal to the splenic flexure. We estimated the area under the receiver operating characteristics curve using c‐statistics to validate the model of this study. We evaluated the two groups by using the absolute standardized differences (ASDs) before and after matching to confirm propensity scoring balance. If ASDs were >10% after matching, then we considered a meaningful imbalance. We used a logistic regression model to estimate the propensity score. Because our current understanding of confounders of CSP outcomes between experienced and trainee groups is limited, we included factors that are reportedly associated with the efficacy and safety of CSP in the propensity score model.

In this study, we collected and analyzed data using a lesion as the unit. In other words, two SSLs occurring in the same patient were analyzed as two independent SSLs. However, SSLs in the same patient could be correlated based on common characteristics, bleeding tendency, degree of colon preparation, and underlying diseases. The Student *t*‐test or Mann‐Whitney U test was used to compare continuous variables, and the Fisher exact test or chi‐squared test was used to compare categorical variables. *p‐*Values <0.05 were considered statistically significant. All statistical analyses were performed using SPSS version 18 (SPSS).

## RESULTS

### Baseline characteristics and outcomes

**FIGURE 2:**
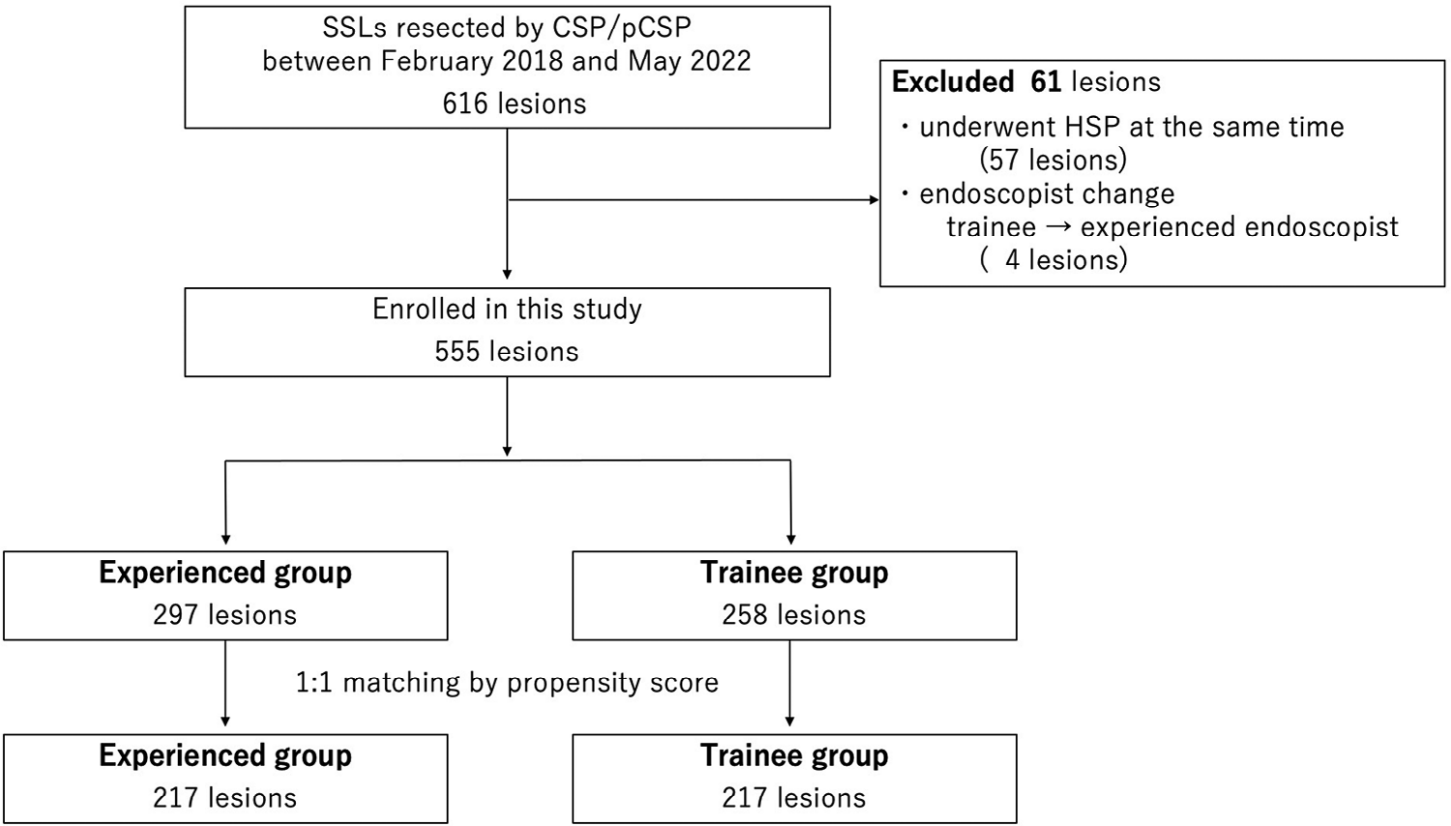
**Flowchart of patient enrollment**. Abbreviations: CSP, cold snare polypectomy; HSP, hot snare polypectomy; pCSP, piecemeal cold snare polypectomy; SSL, sessile serrated lesion.

A flow diagram of patient enrollment is shown in Figure 2 and the baseline characteristics are shown in Table 1. Regarding the baseline characteristics, the trainee group had better colon preparation (84.8% vs. 93.8%; *p <* 0.01 for good‐excellent) and the experienced group had larger lesions (16.3 vs. 12.2 mm; *p <* 0.01 for size), and more previous endoscopic attempts (5.7% vs. 1.6%; *p =* 0.013 for yes). None of the lesions were diagnosed as having cytological dysplasia or as cancer.

**TABLE 1:** Patient and lesion characteristics.

	Baseline cases				Propensity‐matched cases			
	Experienced	Trainee	*p*‐value	ASD	Experienced	Trainee	*p*‐value	ASD
Lesions, *n*	297	258			217	217		
Age, mean (SD)	61.3 (9.4)	60.5 (11.4)	0.82	0.077	61.4 (6.8)	61.2 (7.3)	0.98	0.004
Sex female, *n* (%)	140 (47.1)	124 (48.1)	0.88	0.069	101 (46.5)	104 (48.0)	0.80	0.028
Colon preparation good‐excellent, *n* (%)	252 (84.8)	242 (93.8)	<0.01	0.188	199 (91.7)	201 (92.6)	0.62	0.018
Location proximal colon, *n* (%)	206 (69.4)	175 (67.8)	0.83	0.054	148 (68.2)	146 (67.3)	0.94	0.043
Size, mm, mean (SD)	16.3 (4.8)	12.2 (4.2)	<0.01	0.202	14.9 (3.7)	14.2 (3.2)	0.91	0.059
Morphology type 0‐I, *n* (%)	43 (14.5)	41 (15.9)	0.69	0.041	32 (14.7)	29 (13.3)	0.43	0.071
Previously attempted, *n* (%)	17 (5.7)	4 (1.6)	0.01	0.178	6 (2.7)	4 (1.8)	0.56	0.068
Antithrombotic therapy continuation, *n* (%)	33 (11.1)	34 (13.1)	0.68	0.036	25 (11.5)	25 (11.5)	1.00	0

Abbreviations: ASD, absolute standardized differences; SD, absolute standardized differences; SSL, sessile serrated lesion.

*Note*: Lesion location was classified into two locations: the proximal colon, including the cecum, ascending colon, and transverse colon, and the distal colon, including the descending colon, sigmoid colon, and rectum. The morphology of the lesions was evaluated according to the Paris classification. The term Previously attempted means previous endoscopic attempt including a biopsy of the target lesion.

As for the baseline outcomes, no significant differences in the complete resection rate (99.7% vs. 99.6%; *p =* 0.98) or the adverse event rate (4.4% vs. 3.1%; *p =* 0.82) were seen between the two groups. The en‐bloc resection rate was lower in the experienced group (72.4% vs. 79.1%; *p <* 0.01), and the procedure time was longer in the experienced group (7.9 min vs. 5.7 min; *p =* 0.03). No perforations occurred in either group. Nineteen cases of IPB and two cases of DPPB occurred, all of which were successfully treated using endoscopic clipping. These two patients of DPPB occurred were not receiving antithrombotic therapy. Post‐procedural biopsies detected residual serrated tissue in only two cases (0.36%); the first lesion was 12 mm in size and was located in the ascending colon, and the second lesion was 18 mm in size and was located in the transverse colon. SC was performed at the sites of 461 (83.1%) of the 555 resected lesions at a median of 7 months (range: 3–14 months) after resection. Even the two cases with positive post‐procedural biopsies did not develop any recurrences.

### Characteristics and outcomes after propensity score matching

The characteristics after propensity score matching are shown in Table 1. The c‐statistic for goodness of fit was 0.653 (95% confidence interval 0.607–0.699). Finally, 217 pairs were matched in this study, and the differences in the outcomes of CSP for SSLs ≥10 mm were compared between the two groups (Table 2). Between experienced and trainee groups, the results were the complete resection rate (100% vs. 100%; *p =* 1.00), en‐bloc resection rate (73.2% vs. 75.6%; *p =* 0.24), adverse event rate (3.2% vs. 2.8%; *p =* 0.77), or procedure time (6.2 min vs. 5.9 min; *p =* 0.64).

**TABLE 2:** Clinical outcomes of cold snare polypectomy/Piecemeal cold snare polypectomy (CSP/pCSP) for sessile serrated lesions (SSLs).

	Baseline cases			Propensity‐matched cases		
	Experienced	Trainee	*p*‐value	Experienced	Trainee	*p*‐value
Lesions, *n*	297	258		217	217	
Complete resection, *n* (%)	296 (99.7)	257 (99.6)	0.98	217 (100)	217 (100)	1.00
*EnE*‐bloc resection, *n* (%)	215 (72.4)	204 (79.1)	<0.01	159 (73.2)	164 (75.6)	0.24
Adverse events, *n* (%)	13 (4.4)	8 (3.1)	0.82	7 (3.2)	6 (2.8)	0.77
IPB, *n* (%)	12 (4.0)	7 (2.7)		7 (3.2)	6 (2.8)	
DPPB, *n* (%)	1 (0.4)	1 (0.4)		0	0	
Perforation, *n* (%)	0	0		0	0	
PPS, *n* (%)	0	0		0	0	
Procedure time, min, mean (SD)	7.9 (6.8)	5.7 (6.1)	0.03	6.2 (3.8)	5.9 (3.4)	0.64

Abbreviations: CSP, cold snare polypectomy; DPPB, delayed postpolypectomy bleeding; IPB, immediate postpolypectomy bleeding; pCSP, piecemeal cold snare polypectomy; PPS,post‐polypectomy syndrome; SD, absolute standardized differences SSL, sessile serrated lesion.

*Note*: We defined complete resection as biopsies of the post‐CSP mucosal defect being negative. The procedure time for CSP was measured from the insertion of the snare into the working channel until the completion of the treatment.

## DISCUSSION

SSLs, regardless of their size, have a low risk of containing malignant components.^1^, ^2^ Recently several reports of CSP for SSLs ≥10 mm have been made,^11^, ^12^, ^13^, ^14^, ^15^, ^16^ but all of them were either performed by multiple experienced endoscopists or only one experienced endoscopist. Few of them were performed by trainee endoscopists, and most of them were performed in high‐volume centers, suggesting that the encouraging results might have been partly attributable to the high skill of the endoscopists, who had a lot of experience performing endoscopic treatments such as colorectal ESD.^7^ This suggests that CSP requires dedicated training, such as that for EMR and ESD,^6^, ^7^ and endoscopists may require time to acquire the necessary skills. In summary, previous studies were conducted by experienced endoscopists who had performed many technical procedures, and no comparative studies of outcomes according to the skill levels of the endoscopists have been made. In the present study, we investigated the safety and efficacy of CSP for SSLs ≥10 mm in both experienced and trainee groups to determine whether CSP is a feasible procedure even for trainees, unlike the situation for colorectal ESD, which should be performed by experienced endoscopists only.^7^ This is the only study examining CSP for SSLs that uses propensity score matching to eliminate the effects of potential confounders.

In this study, we removed 555 lesions easily and safely, including 67 lesions in patients receiving uninterrupted antithrombotic therapy. In the baseline cases before propensity matching, the en‐bloc resection rate was significantly lower, and the procedure time was significantly longer in an experienced group. After propensity score matching, in the baseline cases before propensity score matching, the en‐bloc resection rate was lower in an experienced group than in the trainee group because of the larger lesion size. A large lesion size makes it more difficult to perform an en‐bloc resection. Also, in the experienced group, the colon preparation was not as good and pCSP was chosen more often for larger lesions, which may have resulted in the longer procedure time. We believe that the experienced group had a lower en‐bloc resection rate and a longer procedure time for these reasons.

In this study, the five endoscopists were trainee endoscopists who were not yet certified by the Japanese Society of Gastrointestinal Endoscopy. In addition, they had never performed colorectal ESD before, but they were able to perform the CSP procedure in most cases without being taken over by experienced endoscopists. We had four cases that were taken over by experienced endoscopists, but these lesions were at the beginning of the study (during the first 2 months) and were taken over as a precaution. All 4 lesions were less than 20 mm in diameter and were resected en‐bloc by experienced endoscopists with no adverse events. The current ESGE (European Society of Gastrointestinal Endoscopy) guidelines recommend that colorectal ESD be performed by trained endoscopists in tertiary care facilities.^21^ The results of this study showed that there was no difference in the outcomes of CSP for SSLs when performed by experienced and trainee groups, suggesting that the CSP technique, unlike colorectal ESD, does not require a high level of skill. In addition, the risk of perforation without electrocautery is low, suggesting that CSP for SSL ≥10 mm can be performed even in non‐tertiary care facilities.

A strength of our study is its relatively large sample size, compared with the sample sizes of published studies comparing the efficacy and safety of CSP for SSLs ≥10 mm. We enrolled a lot of lesions and were able to match 217 pairs (434 lesions) for analysis. The present study is also the only one to compare multiple endoscopists and to use propensity score matching to eliminate the effects of potential confounders among endoscopists.

The present study has several limitations. First, although the subjects of this study were prospectively enrolled in our database, detailed data regarding the patients were retrospectively collected from medical records. In this study, we were able to apply propensity score matching to ensure comparability between the two groups and to examine the efficacy and safety of CSP for SSLs ≥10 mm. Second, since our study was conducted at a single Japanese endoscopy center, further multicenter studies are needed. Third, although we did not compare recurrence in this study, the median follow‐up period was 7 months; further long‐term outcome data are therefore needed because SSLs progress slowly. In addition, it is necessary to consider the size that affects the recurrence in the future. This is because PSM was used in this study, but there was a difference in the size of the resection specimen among both groups before PSM. In actual clinical practice, depending on the skill of the endoscopists, we have to consider which size is a safe and useful CSP procedure for SSLs. Fourth, the influence of the learning curve of CSP in the experience of endoscopists cannot be removed. However, as mentioned in the strength of this study, CSP was performed by as many as 13 endoscopists, and the difference between endoscopists was also considered a confounding factor. Finally, to avoid confusion as to which procedure was responsible for DPPB, we excluded patients who underwent HP and CSP during the same session. However, according to previous reports, DPPB after CSP occurred in 0%–1.8 % of cases.^22^ DPPB occurred in only two cases in the present study. Therefore, the risk of DPPB occurring in patients undergoing HP and CSP during the same session is considered to be negligible.

In conclusion, we have demonstrated the safety and efficacy of CSP for SSLs ≥10 mm between experienced and trainee endoscopists.

## CONFLICT OF INTEREST STATEMENT

None.

## ETHICS STATEMENT

All procedures were performed at the NTT Medical Center Tokyo. Written informed consent was obtained from each patient. This study was approved by the ethics committee of the NTT Medical Center Tokyo (No. 18–81).
